# The DMP atlas for North Rhine-Westphalia, Germany: results of the disease management programmes on district level

**DOI:** 10.1007/s43999-026-00088-2

**Published:** 2026-02-24

**Authors:** Sabine Groos, Jens Kretschmann, Arne Weber, Michaela Assheuer, Bernd Hagen

**Affiliations:** Central Research Institute of Ambulatory Health Care in Germany, Department of Evaluation and Quality Assurance, Cologne, Germany

**Keywords:** Atlas, Disease management programmes, DMP, Health care quality, Regional variation

## Abstract

Disease Management Programmes (DMP) in Germany were initiated to enhance the quality of ambulatory health care for specific chronic diseases. They aim to prevent the progression of the disease and comorbidity as well as to improve quality of life. It is the purpose of this paper to describe the contents of the web-based DMP atlas for North Rhine-Westphalia. The atlas provides a comprehensive overview of the regional DMP results, including outcomes at district level. In 2024, these analyses encompassed data of approximately 1.8 million patients. Quality of care is displayed by quality indicators for type 2 and type 1 diabetes, coronary heart disease, bronchial asthma, chronic obstructive pulmonary disease, breast cancer and osteoporosis. Outcomes are displayed at district level and can be stratified by age, sex, GPs’ or specialists’ care within the DMPs, the documentation of selected comorbid conditions and DMP participation period. Furthermore, information on comorbidity, health parameters, risk factors and medication are displayed. Again, these can be stratified by age and sex. The DMP atlas allows to identify areas with lower quality of care and subgroups of particularly vulnerable patients. Consequently, it can be seen as a contribution to the further development and specification of DMPs and ambulatory health care in Germany.

## Introduction

Disease Management Programmes (DMPs) were initiated in Germany in 2002 to improve the quality of ambulatory health care for individuals with specific chronic diseases [1]. Hence, they provide a structured framework for high-quality care and clearly defined quality indicators against which care can be evaluated. Furthermore, DMPs facilitate benchmarking by enabling individual practices to compare their results with those of other practices through detailed feedback reports.

DMP contracts are concluded at the federal state level between the respective associations of statutory health physicians and statutory health insurance funds. To date, in the German federal state of North Rhine-Westphalia (NRW) DMP contracts have been established for type 2 and type 1 diabetes, coronary heart disease, bronchial asthma, chronic obstructive pulmonary disease, breast cancer and, lately, osteoporosis. The number of people in Germany covered by at least one DMP is currently 7.3 million [2]. Further DMPs are expected to be implemented, e.g. concerning adiposity in children and adults.

The general framework and objectives of the DMPs are determined by the Joint Federal Committee [3]. In general, DMPs aim to prevent the progression of the underlying disease as well as comorbidity and to improve quality of life of the chronically ill people. To enhance a structured and evidence-based treatment in Germany, they are based on the current German national health care guidelines for the particular disease. To evaluate the quality of care, several indicators are defined for each DMP (Table 1).

**Table 1:** Indicators of quality of care in the DMPs

Indicator	DMP
Participation in training courses, self-management	AB, CHD, COPD, OP, T1D, T2D
Smoking	AB, CHD, COPD
Smoking cessation	AB, CHD, COPD
Physical training	BC, CHD, COPD
Blood pressure in patients with arterial hypertension	CHD, T1D, T2D
Respiratory medication therapy and use of devices	AB, COPD
Antiplatelet therapy	CHD, TD1
Beta blocker therapy after myocardial infarction	CHD
Lipid-lowering therapy	CHD
Osteoporosis medication therapy	OP
Endocrine therapy	BC
Cardiotoxic therapy	BC
Antidiabetic medication therapy	T2D
HbA1c levels	T1D, T2D
Severe hypoglycemic events	T1D, T2D
Inpatient treatment	T1D, T2D
Emergency situations and exacerbations	AB, COPD
Assessment of forced expiratory volume in 1 s	AB, COPD
Assessment of renal function	T1D, T2D
Assessment and therapy of feet state	T1D, T2D
Assessment of insulin injection site	T1D
Assessment of albumin-to-creatinine ratio in urine	T1D
Assessment of ophthalmological function	T2D
Angina pectoris symptoms	CHD
Control of asthma symptoms	AB
Bone fractures and fall prevention	OP
Metastases and lymphoedema	BC
Osteodensometry	BC

AB: bronchial asthma, BC: breast cancer; CHD: coronary heart disease; COPD: chronic obstructive pulmonary disease; OP: osteoporosis; T1D: type 1 diabetes; T2D: type 2 diabetes

Since the beginning of the DMPs, the Central Research Institute of Ambulatory Health Care (Zi) has been assigned to evaluate and report on the quality of care within the DMPs in several regions of Germany. To date, the Zi accounts for the evaluation of the DMP in four federal states: Baden-Wuerttemberg, Bremen, Lower Saxony and North Rhine-Westphalia, the latter comprising the two regions of North Rhine and Westphalia-Lippe. By the end of 2024, these encompassed a total of 0.934 million, 0.072 million, 0.811 million, 1.019 million and 0.798 million patients enrolled in at least one DMP, respectively.

The Zi’s evaluation of the DMPs comprises annual quality reports with open access [4, 5] as well as semiannual benchmarking feedback reports to the practices participating in the DMP, depending on the mandate of each federal state.

The Zi’s quality reports cover health parameters such as HbA1c values for people with diabetes, FEV1 values for people with respiratory diseases and information on comorbidities, medical treatment and check-ups. Furthermore, the above mentioned indicators are analysed to picture the quality of care as well as its changes over time. Certain information is stratified by relevant patients’ characteristics such as sex, age or comorbidity.

The DMP atlas was inspired by the already existing Zi’s health care atlas Germany [6] and stimulated by the Diabetes Surveillance project [7]. This project, coordinated by the Robert Koch Institute, developed periodic indicator-based health reporting on diabetes in Germany. In 2019, the Zi launched the first web-based interactive DMP atlas for the federal state of North Rhine-Westphalia [8] There were two main reasons for this.

On the one hand, the scope for the presentation of DMP outcomes within the quality reports is limited due to the extensive documentation requirements, parameters and quality indicators inherent to the various DMPs. Furthermore, the results are found to vary according to subgroups of patients, e.g. by sex and age. Additionally, the results show regional variation between districts and cities. Thus, it was impossible to meet the needs for detailed information by not inflating the quality report beyond reasonable extent at the same time.

On the other hand, the quality reports are primarily addressed to physicians, researchers, stakeholders and health policymakers. The digital provisioning of information about the DMP outcomes is supposed to broaden the spectrum of people interested in the data.

The atlas provides a comprehensive overview of the regional DMP results, including outcomes at district level within the respective regions. The purpose of this paper is to present the DMP atlas and to give some insight into the information provided by it. For practical reasons, the examples will be limited to information on the DMPs for people with type 2 diabetes, coronary heart disease or chronic obstructive pulmonary disease in 2023. Further details can be found at. http://www.zi-dmp.de/dmp-atlas_nrw.

## Technical information and data

Patients enrolled in a DMP are expected to attend regular medical consultations, usually on a quarterly basis. Medical data are documented electronically using standardised forms. These records are exported to a trusted third party data center, where they are pseudonymised and subsequently transmitted to the Zi.

Information displayed in the DMP atlas are derived from pseudonymised DMP documentation data at patient’s level. Geographic allocation to districts and regions is not based on patients’ place of residence, since this information is not available in DMP documentation for privacy reasons, but on practice locations.

The DMP atlas presents precalculated results with different levels of aggregation but does not display data at patient’s case level. However, given that proportions can be confounded by low numbers and for privacy reasons regarding the practices, information is not displayed if it concerns less than 30 patients or three practices per district. The DMP atlas is updated annually.

## Contents of the DMP atlas

The following text presents the DMP atlas through selected sample pages. The figures included as examples illustrate the DMP results at both the regional and individual district levels. They provide a snapshot of the results from 2023. As the atlas is updated regularly, all results presented are subject to ongoing changes. By December 2025, the atlas pages have been updated with data up to the end of 2024.

In 2023, a total of approximately 1 077 100, 456 500 and 192 100 patients were enrolled in the DMPs related to type 2 diabetes (T2D), coronary heart disease (CHD) and chronic obstructive pulmonary disease (COPD) in North Rhine-Westphalia, respectively.

### Dashboard reports on quality of care

**Fig. 1:**
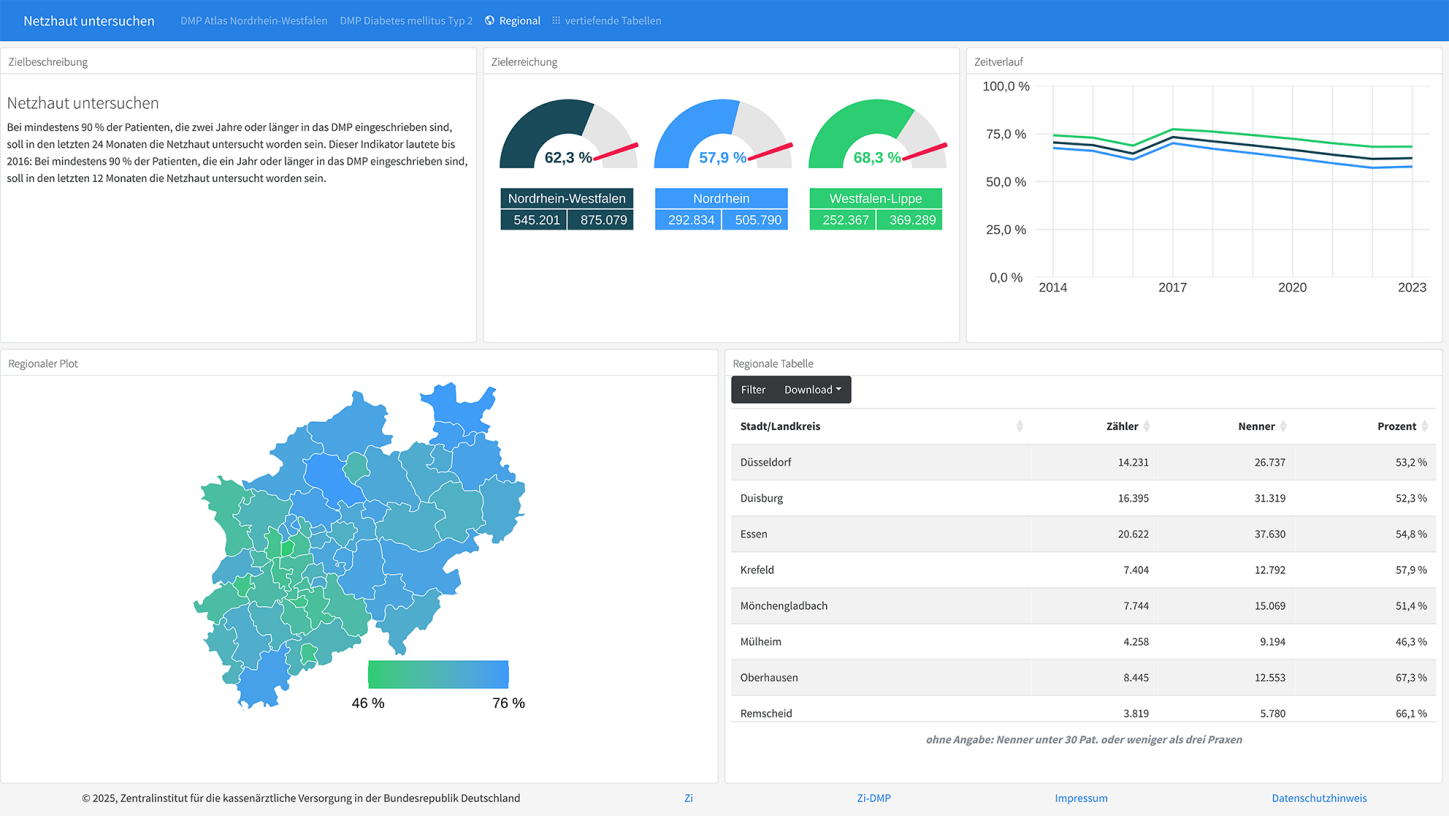
DMP T2D – Dashboard report of regular retinal check-ups in North Rhine-Westphalia. Notes: Description of Fig. 1, DMP T2D, quality of care indicator “Retinal check-up”, in English: Upper left segment – “Definition of quality target”, “At least 90% of patients enrolled in the DMP for two years or more should have undergone a retinal examination with in the past 24 months. Until 2016, the target was that at least 90% of patients enrolled for one year or more should have had their retina examined in the past 12 months.”. Upper center segment – “Goal achievement”. Upper right segment – “Time course of goal achievement”. Lower left segment – “Regional map”. Lower right segment – “Regional table”, rows represent cities and districts, columns show numerator, denominator, and percentage; results of districts with less than 30 cases and/or less than three practices are not presented

The first objective of the atlas is to provide an overview of the quality of care within the DMPs regarding the defined quality indicators. Therefore, dashboard reports are available separately for each quality indicator in each DMP. For instance, Fig. 1 shows the frequency of retinal check-ups which should be conducted every two years in people with type 2 diabetes.

In the state of North Rhine-Westphalia, 62.3% of the patients underwent such a retinal check-up (data status in 2023). Looking at the two areas of North Rhine and Westphalia-Lippe separately, data reveal prevalences of 57.9% and 68.3%, respectively. On the upper left, a definition of the indicator is presented along with a description of any changes in the definition over time.

The latter is an important issue when looking at the development of prevalences of retinal check-up over time (upper right). Evidently, there is a declining trend over the past few years. But there also seems to be a hump in 2017. As noted in the indicator’s description (to date, only available in German), rather than being a real increase in control frequencies, this hump is due to a modified definition of the quality indicator. Until 2016, retinal check-ups have been supposed to be conducted every year, afterwards they became mandatory every second year.

Moreover, quality of care, based on the respective indicator, is mapped on district level. On the lower left side, variations of frequencies of retinal check-up in the different districts are displayed. They vary from 46% to 76%. Frequencies for each district can be displayed both, interactively within the map as well as in the table at the lower right side. Absolute and relative numbers concerning the quality indicators are given for all regions and districts.

The atlas also allows deeper insight into the quality of health care regarding subgroups of patients, stratified by age, sex, GPs’ or specialists’ care within the DMPs, the documentation of selected comorbid conditions and DMP participation period. All tables can be displayed both, univariately and cross-tabulated for several strata.

### Reporting of comorbidity and selected parameters

Within the DMP documentation additional information about comorbidity, health parameters, risk factors and medication are available. Thus, the second objective of the atlas is to provide more details about and a deeper insight into the health conditions of the patients enrolled in the DMPs.

Depending on the information documented in each DMP, several comorbid conditions and parameters are displayed. An overview is given in Table 2.

**Table 2:** Comorbidity, health parameters, risk factors and medication displayed in the DMP atlas

Comorbidity	DMP
Acute coronary syndrome	CHD
Arterial hypertension	AB, CHD, COPD, T1D, T2D
Bronchial asthma	CHD, COPD, T1D, T2D
Chronic heart failure	AB, CHD, COPD, T1D, T2D
Chronic obstructive pulmonary disease	AB, CHD, T1D, T2D
Coronary heart disease	AB, COPD, T1D, T2D
Diabetes mellitus	AB, CHD, COPD
Diabetic foot syndrome	T1D, T2D
Diabetic neuropathy and amputation	T1D, T2D
Diabetic nephropathy and dialysis	T1D, T2D
Diabetic retinopathy and blindness	T1D, T2D
Dyslipidemia	AB, CHD, COPD, T1D, T2D
Myocardial infarction	CHD, T1D, T2D
Peripheral arterial occlusive disease	AB, CHD, COPD, T1D, T2D
Stroke	CHD, T1D, T2D

Health parameters, risk factors, medication	DMP
Angina pectoris symptoms	CHD
Antidiabetic medication	T2D
Asthma symptoms	AB
Blood pressure	CHD, T2D
Forced expiratory volume in 1 s	AB, COPD
HbA1c	T1D, T2D
LDL cholesterol level	CHD
Smoking	T1D, T2D
State of feet	T2D
State of insulin injection site	T1D
Albumin-to-creatinine ratio in urine	T1D
Height, weight and adiposity	AB, COPD, T1D, T2D

AB: bronchial asthma, CHD: coronary heart disease; COPD: chronic obstructive pulmonary disease; T1D: type 1 diabetes; T2D: type 2 diabetes

**Fig. 2:**
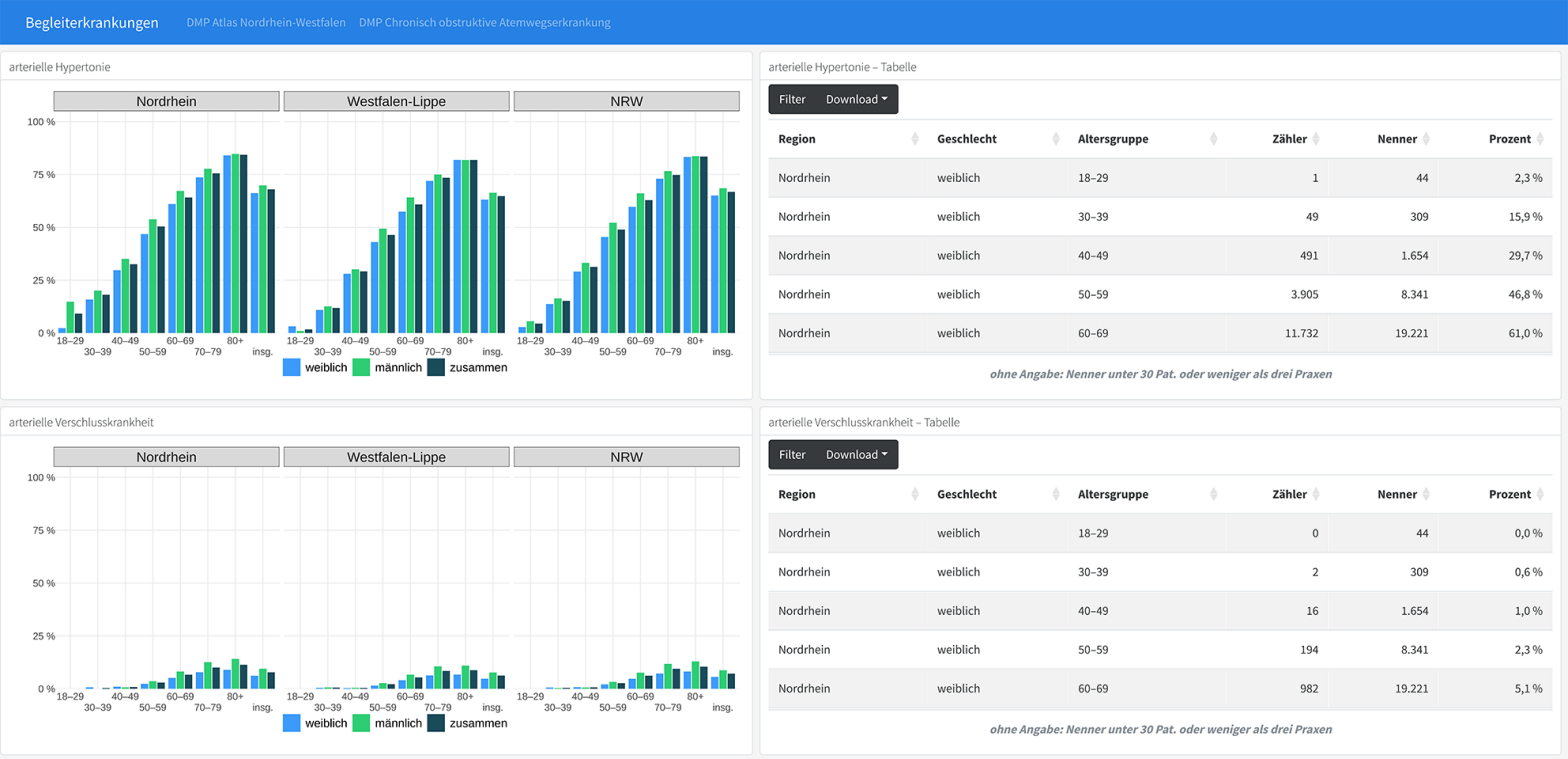
DMP COPD – Prevalences of comorbid conditions stratified by age, sex and region (excerpt). Notes: Description of Fig. 2, DMP COPD, prevalence of comorbid diseases, in English: Graphics: Examples for arterial hypertension and peripheral arterial occlusive disease, stratified be age group (years) and sex (female, male, total). Table: Rows represent regions, columns show sex, age group, numerator, denominator, and percentage

Figure 2 shows an example of prevalences of comorbid conditions in people with COPD. Stratified by region, age group and sex, prevalences of arterial hypertension and peripheral arterial occlusive disease are shown. In general, they increase with age, are higher in males and show similar distributions in both regions, North Rhine and Westphalia-Lippe.

**Fig. 3:**
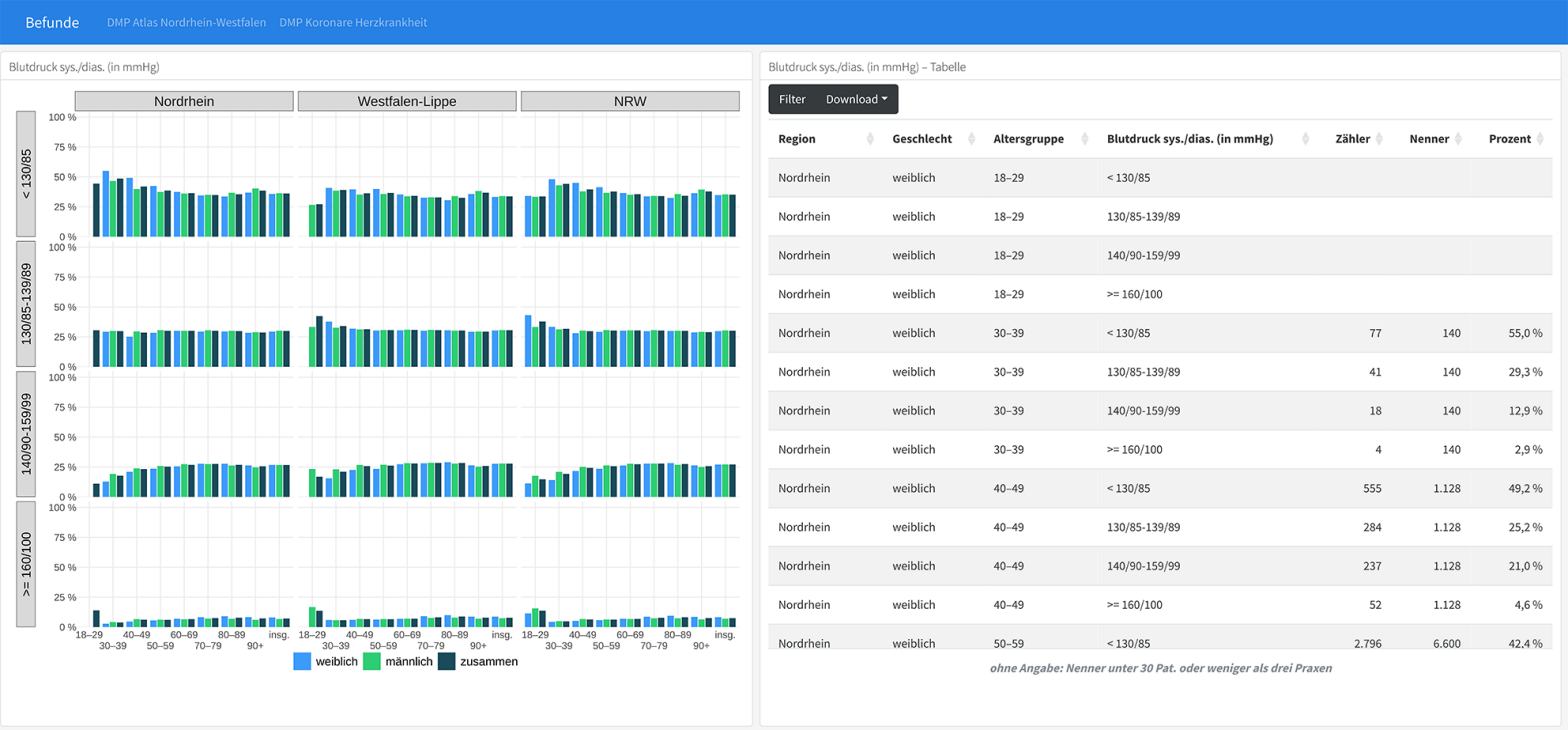
DMP CHD – Blood pressure levels stratified by age, sex and region. Notes: Description of Fig. 3, DMP CHD, blood pressure levels, in English: Graphics: Blood pressure levels stratified by age group (years) and sex (female, male, total). Table: Rows represent regions, columns include sex, age group, blood pressure level, numerator, denominator, and percentage

Furthermore, Fig. 3 displays blood pressure levels in people with CHD, stratified by region, sex and age group. Few patients suffer from very high blood pressure, with a slight increase with age and defined differences neither between sexes nor between the two regions. In both figures, absolute and relative numbers are given. Absolute numbers refer to the number of cases, while relative numbers represent the proportion of blood pressure levels within each age group and sex.

## Limitations and strengths of the DMP atlas

Limitations are mainly due to the nature of documented data within the DMPs. First, geographical allocation is only possible on the basis of practice locations. However, given the predominantly GP-based nature of care within the DMPs, a close geographical proximity between the respective GP practice and the DMP patient’s place of residence can be assumed in most cases. Consequently, the probability of commuting across district borders is considered to be low.

Second, as described above, due to privacy reasons presentation of results in the DMP atlas is restricted to findings from a minimum of three DMP practices or data from a minimum of 30 DMP cases per district. These restrictions might have substantial impact on the capacity to display DMP results at district level in certain subgroups, e.g. GPs’ versus specialists’ care.

Third, quality indicators and DMP documentation are subject to continuous change. New indicators may be introduced, old ones be abandoned and indicator definitions change. Concurrently, the accuracy of DMP documentation can fluctuate. This limits the comparison of outcomes over time.

The atlas’s main strength is its detailed small-scale presentation of all DMP results. This enables meaningful analysis at the district level across all DMPs. Consequently, districts demonstrating particularly strong performance can be distinguished, as well as those exhibiting comparatively lower outcomes.

## Conclusion

The regionalised and stratified presentation of the DMP outcomes in the DMP atlas reveals a considerable degree of variation. Substantial disparities are observed not only between individual districts but also among patient groups concerning age, sex, comorbidity, GP’s or specialist’s care or DMP participation period. Furthermore, the development of quality of care within the DMPs can be tracked over time.

The DMP atlas displays these outcomes in a transparent, interactive and up-to-date manner. To our knowledge, no other comparable regional and stratified presentation of information on the DMPs exists in Germany. As a significant enhancement, future development of the DMP atlas will include the interquartile range of results at the regional level.

The Joint Federal Committee has stipulated that DMP data may only be used for quality assurance and evaluation by health insurance funds or associations of statutory health insurance physicians [3]. There are currently no plans to make the data publicly available. However, a detailed analysis of the DMP results at the level of individual districts is possible within the framework of regular DMP quality assurance, as is, for example, their use for creating feedback reports, reminders or quality circle-related analyses.

Like the Zi’s health care atlas [6], the DMP atlas presents outcomes in cartographical and tabular manner at district level. However, they rely on different databases: billing data on the one hand and DMP documentation data on the other. Marked regional variation can be observed in both.

Moreover, many other registries and atlases on ambulatory health and health care exist in Europe and beyond [e.g. 9–21]. They are similar to the DMP atlas in some aspects, but differ in others, depending on their purpose, data sources, focus, data currency and time frame. It is beyond the scope of this paper, to discuss this in detail.

As stated in the introduction DMPs were implemented to improve the quality of care for people with chronic diseases. However, differences exist both in the age- and sex-specific conditions of a disease – particularly concerning its progression and severity – and in the medical care structures within a region. This can be seen as a chance to analyse underlying causes, to improve the quality of care, to adapt health policies and to enhance further research.

For instance, the DMP atlas allows to identify areas with lower quality of care and to develop research on the underlying factors like socio-demographic or infrastructural causes. In consequence, ideas on how health care can be improved in districts with below-average quality of care can be developed. In more detailed analyses, results for large cities can even be disaggregated to the level of individual city districts. In a previous pilot study, the Zi plotted DMP outcomes in patients with type 2 diabetes against area level deprivation within the Ruhr metropolitan region [22].

In conclusion, the DMP atlas can be seen as a contribution to the further development and specification of DMPs and ambulatory health care in Germany. It provides information on the extent to which DMP results vary between districts and enables the identification of areas with particularly high and low quality of care.

## Data Availability

Data sharing is not applicable to this article as no datasets were generated or analysed during the current study.
